# The Effect of Hydrocolloids on Penetration Tests and Syneresis of Binary Gum Gels and Modified Corn Starch–Gum Gels

**DOI:** 10.3390/gels9080605

**Published:** 2023-07-26

**Authors:** Hong-Ting Victor Lin, Jenn-Shou Tsai, Hsiao-Hui Liao, Wen-Chieh Sung

**Affiliations:** 1 Department of Food Science, National Taiwan Ocean University, Keelung 202301, Taiwan; hl358@mail.ntou.edu.tw (H.-T.V.L.); tsaijs@mail.ntou.edu.tw (J.-S.T.); 2 Center of Excellence for the Oceans, National Taiwan Ocean University, No. 2, Pei-Ning Road, Keelung 202301, Taiwan; 3 Weichuan Foods Corporation, New Taipei City 22102, Taiwan; sammi_liao@weichuan.com.tw

**Keywords:** gellan gum, gelatin, agar, synersis, modified corn starch

## Abstract

The interactions among agar, gellan gum, gelatin, and modified waxy corn starch in the formation of mixed gels were examined in five different ratios. Binary hydrocolloid gels were prepared using three ingredients: two hydrocolloids (total hydrocolloid concentration: 0.5 wt%, ratios of mixture: 0/0.5, 0.1/0.4, 0.2/0.3, 0.3/0.2, 0.4/0.1, and 0.5/0) and water. The textural properties of the hydrocolloid gels were studied by measuring the gel strength, rigidity, breaking force, breaking point, and syneresis as functions of the mixing ratio. The higher syneresis percentage of binary modified waxy corn starch and gum gels than that of mixed gum gels after cold storage was mainly due to the retrogradation of amylopectin. Agar was shown be the most influential with regards to increasing the gel strength, breaking force, and rigidity among the three kinds of gum, while gellan gum was more effective against syneresis than agar and gelatin for storage periods of 7 and 14 days. In the mixed gels, a dramatic increase in the breaking point from 0 to 0.5% was only exhibited for gellan gum. The results provided useful information, including gel strength, rigidity, breaking force, breaking point, and syneresis, for gum and modified corn starch ingredients selected from refrigerated binary gum gels such as pudding for food product development.

## 1. Introduction

Hydrocolloids are primarily responsible for the functional properties and the qualities of many processed foods. Processed foods normally contain mixtures of hydrocolloids for starch, imparting structural functions and providing attractive sensations [1,2]. Research on hydrocolloid interactions in gels is important for the improvement of traditional foods, the development of novel processed foods, and in contributing functional properties of food systems [3,4].

Gellan gum is an anionic deacetylated exocellular polysaccharide produced by microbe *Pseudomonas elodea* with repeating tetrasaccharide (1,3-β-D-glucose, 1,4-β-D-glucuronic acid, 1,4-β-glucose,1,4-α-L-rhamnose) units containing one carboxyl side group [5,6]. The linear anionic heteropolysaccharide of gellan can induce a high viscosity and forms a double helix in aqueous solutions. Gellan gum was approved for food use by the FDA in 1992. Gellan gum develops a reversible temperature-dependent gel and is extremely effective at low use levels in forming gels [7]. Gellan gum forms double helices and aggregates in junction zones, which form a three-dimensional network with divalent or monovalent cations. The native gellan polymer is high in acyl groups. The acyl groups of gellan gum are deacylated and hydrolyzed into a form of low acyl gellan when exposed to alkalis at high temperatures. Sanderson et al. [8] noted that high acyl gellan gels are elastic and non-brittle, with a texture like the mixed gels of xanthan gum and locust bean gum.

Gelatin is the main protein gelling agent obtained by the partial hydrolysis of collagen derived from the collagen present in connective tissues, pig or cow skins, and bones [9,10]; gelatin has a lower melting and gelation temperature, a longer setting time, and it requires a higher concentration compared to gellan gum [11]. Gelatin dissolves in hot water and forms a gel when cooled. The triple helices unravel and dissolve as random coils when solid gelatin is heated. Junction zones are formed by small segments from two or three polypeptides returning to the collagen triple helix-like structure when the gelatin solution is cooled. Gelatin gels are very elastic. Gelatin can form gels over an extensive range of concentrations so that it can be used in a broad range of products, such as mousses, fruit gum, jellies, and marshmallows [2,12].

Agar is a cell wall polysaccharide extracted from the algae Gracilariaceae and Gelidiaceae families in water at temperatures above the melting points of their gels. Extraction may be done under acidic, neutral, or basic conditions, the selection being controlled by the type of agarophyte and the qualities required in the gel [13]. Agars are linear polysaccharides made up of alternating 1,3-linked β-D-galactopyranose and 1,4-linked 3,6-anhydro-α-L-galactopyranose units [14]. Agarobiose is the basic disaccharide structural unit of all agar polysaccharides. Agar can be fractionated into agarose and agaropectin. Agarose is a neutral polysaccharide with the greatest gelling capability. Agaropectin contains all the charged polysaccharide components; some of residues have been replaced by pyruvic acid ketal, 4,6-O-(1-carboxyethylidene) D-galactopyranose, or by methylated or sulphated sugar units [14]. The ratio of agarose to agaropectin varies depending on the alga source and affects the physicochemical, mechanical, and rheological properties of agar [15]. When heated, the agar molecules are homogenously distributed throughout the aqueous solution in a random coil conformation. When the solution is cooled, agar molecules associate with each other, forming an elastic and turbid double-helix cross-linked network [16]. Because agar can form a very hard gel at very low concentrations and does not require the presence of any cation for gelation, agar has been broadly used as thickeners, stabilizers, and gelling agents in food manufacturing.

A mixture of multi-component gels provides better control in processed food products than a single component system. The synergistic effect of single-phase mixed solutions of polysaccharides is due to the excluded volume effect of macromolecules [17]. The excluded volume effects enhance the gelation of hydrocolloids. The rate of gelation is faster, and the minimal concentration required for gelation is lower than that of each individual polysaccharide when dealing with incompatible polysaccharides in mixed solutions [18].

Starch is a major storage energy reserved in plants such as rice, maize, potatoes, and wheat. Starch is the most studied natural polymer in an insoluble granular form. Starch consists of two different glucose polymers: primarily branched (amylopectin) and linear (amylose) chains of molecules. Amylopectin is insoluble in hot water, and it is essentially a highly branched glucose molecule in waxy genotype starch. On the other hand, amylose is soluble in hot water, and it is a primarily crystalline linear glucose molecule with a few branches. When starch granules are heated in the presence of water at temperatures higher than 60 °C, the starch granules swell and rupture, accompanied by an increase in viscosity by dissociating double-helix amylopectin hydrogen bonds. It is also known as the gelatinization of starch. Gelatinized starch imparts mouthfeel and body to a food product. Starches can also interact with hydrocolloids [19]. Since hydrocolloids and starches usually coexist in many processed foods, their interaction affects the textures of these foods. The synergistic interaction occurring between hydrocolloids and starches supports a significant increase in the viscosity of the starch paste [20]. Studies on starch–gum systems have exhibited a synergistic effect, culminating in a much higher viscosity of the combination compared to starch or gum alone [21]. Texture is an important characteristic of starch gel quality, and the addition of a stabilizer acting as a gelling agent or thickener, such as gelatin, agar, or other polysaccharides, has been reported to give good stability and pleasant texture [22,23]. When the gelatinized starch is stored at a refrigerated temperature, it undergoes a recrystallization of the amylopectin and amylose molecules. This is also known as the retrogradation of starch.

By binding hydrocolloids with water to diminish the water flow in the matrix space, a resultant resistance to syneresis occurs, as well as a smooth sensation in the mouth [24,25]. Upon cooling, the water can be easily expressed from the starch–gum network, another phenomenon also known as syneresis. This occurrence is usually viewed unfavorably, as it may lead to product deterioration. The extent of syneresis is directly related to the tendency of starch to retrograde [26]. Much less work has been carried out on the effects among gelatin, agar, gellan gum, and modified waxy corn starch on penetration tests and the syneresis of binary hydrocolloid gels. A mixture of design experiments was used to evaluate the interactions among agar, gellan gum, gelatin, and modified waxy corn starch in the formation of a mixed gel structure as a function of different mixing ratios. In addition, the effect of the binary gel ratio during the storage time on improving the textural properties and minimizing the syneresis of binary gels investigated at a refrigerated temperature could be used for gel foods such as pudding for formulation in obtaining a desirable texture in the future.

## 2. Results and Discussion

### 2.1. Measurement of Textural Properties and % Syneresis of Mixed Gels

The textural properties of gelatin/agar gels at various gum concentrations, as determined by the penetration tests, are summarized in Table 1. The addition of agar to gelatin generally resulted in significant (*p* ≤ 0.05) increases in the breaking force, gel strength, and rigidity of mixed gels. However, the gel strength of mixed gels increased more sharply at lower agar ratios. Gel strength was defined as the force either at the point where the slope of the curve changed suddenly or at the first peak in the force deformation curve. The rigidity of the mixed gels was calculated as the initial slope of the force–deformation curve. Table 1 also shows that, for the agar addition, the breaking point of the mixed gels increased with an increase in the gelatin concentration. The breaking point was estimated as the distance between the first touch of the probe on the gel surface and the failure point. The current results showed that agar imparted a firm and brittle texture to the mixed gel body; gelatin provided a soft and elastic texture to the gels. The results indicated that gelatin gel exhibits a delayed breaking point for agar mixed gels. On the other hand, gelatin–agar binary gels are less brittle compared to agar gel alone for developing less brittle type gels when the binary gel breaks inside the mouth. The fracture energy of gelatin–agar binary gel shows a decreasing trend with the gelatin ratio increase. Gelatin can be used to prepare a chewable binary gel that stays in the mouth for a longer duration without easy disintegration, while agar is suitable for developing easy-to-bite gels. It could also be concluded that the gel strength of gelati–agar mixed gel was mainly due to the network structure of agar rather than the triple-helix-like structure of gelatin. The combinations of gelatin and agar have created new opportunities for food product development [27]. A combination of gelatin and agar considerably increased the degree of firmness compared to gelatin alone, and the melting point of the mixed gels increased to 80 °C. Schrieber and Gareis [27] reported that the melting point temperature of fruit gummies could be increased to about 50 °C, making the fruit gummies suitable for marketing in tropical climates. When considering more complicated food gels like milk pudding, its emulsified milk fat property is very likely to be impacted by the above mechanical and physical properties. The relative contributions of the different ingredients, including milk, sugar, cream, and eggs, combined in complicated food gels need explained well in the future. The consideration of more complicated model binary food gels is therefore necessary to progressively fill in the gap with real food gels and to obtain a fundamental understanding of the sensory perceptions and mouth chewing process. The emulsion-filled gels of gelatin and agar were shown to be promising in this way. This binary gum gel offers different formulation ratios that make it possible to obtain various mechanical and structural characteristics consistent with real food gels.

**Figure 1:**
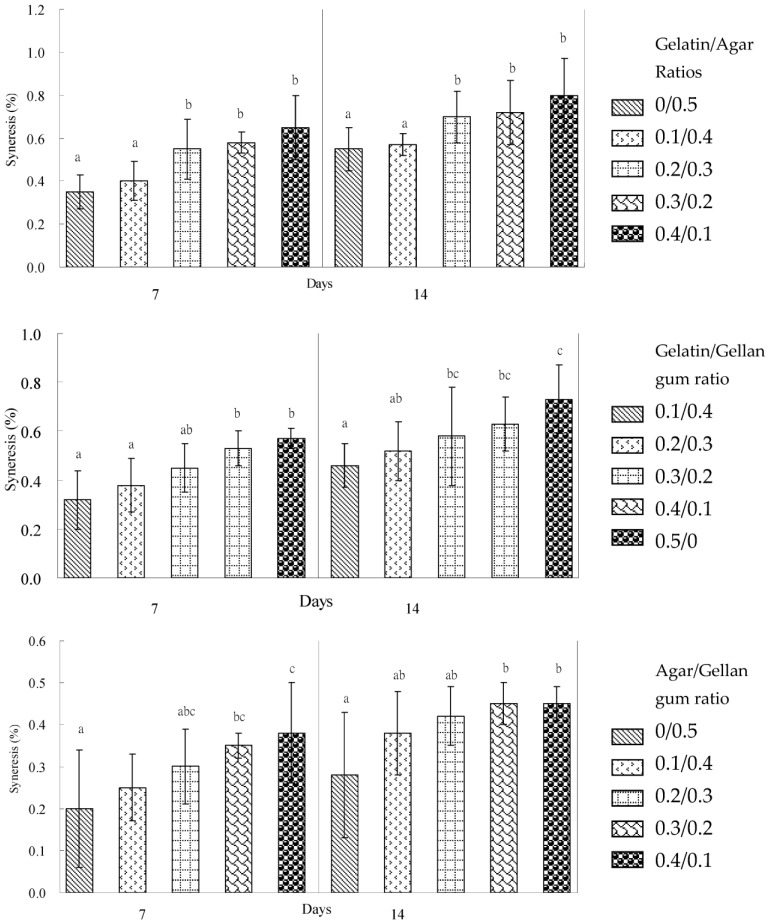
Syneresis of binary mixed gels stored at 4 °C for 7 days and 14 days. Data with different letters for each storage day represent significant differences at *p* < 0.05.

The effect of agar and gelatin on the syneresis percentage in composite gels is presented in Figure 1. Refrigerating agar for periods of 7 and 14 days at the 0.5% and 0.4% ratios significantly (*p* ≤ 0.05) reduced the syneresis percentage of gelatin–agar gels. Mixed gels with agar concentrations of less than 0.3% had a high syneresis value (>0.55%). Agar gel alone is brittle, and it has high melting and gelling temperatures but low syneresis in the 0.4% and 0.5% ratios. Gelatin gel alone at these ratios is soft, with low melting and gelling temperatures. Gelatin forms elastic, flexible, and soft gels. Neither agar or gelatin alone provide a soft and low syneresis food gel.

The effect of different ratios of gelatin and gellan gum on the penetration testing of binary gels is recorded in Table 1; it shows that the addition of gellan gum to gelatin can increase the gel strength, breaking force, and the breaking point of mixed gels. Papageorgiou et al. [28] demonstrated an increase in the gel strength of 5% gelatin gel upon the addition of 0.5% gellan gum. Gelatin is one of the most popularly used types of protein hydrogel on food additives. However, the increase in gel strength and breaking force of the gelatin–gellan mixture in gellan gum (Table 1) was less obvious than the gelatin–agar mixture (Table 1). The results of this study implied a weak interaction between gelatin and gellan when calcium ion was not added. A natural water-soluble polymer is a high acyl gellan containing O-5-acetyl and O-2-glyceryl groups on the (1→3)-linked glucose residue [29]. High acyl gellan gels are very soft, since the bulky acetyl and glyceryl groups hinder the close association between gellan polymer chains in a double-helix formation and prevent the compact packing of the cross-linked double helix [29]. High acyl gellan gum is often used in combination with other gelling agents or alone to produce a wide variety of desirable textures for the food industry. Their textural properties cannot be easily measured in compression tests. This result demonstrated high acyl gellan gum at low use levels in forming soft, non-brittle, very elastic, and fluid gels. The double helix of gellan gum is cohesive because of physical forces, van der Walls forces, and hydrogen bonding. This shows that, when the high gel strength and breaking force of gelatin gel is required, the addition of agar is effective, especially for gelatin gels.

Nevertheless, Table 1 demonstrates that, in cases where the breaking point of composite gels is considered important, the addition of gellan gum is appropriate, particularly for ingredient consideration. Although the penetration tests did not exhibit the shear-thinning behavior of fundamental gel mechanical properties, these empirical results might provide specific functional properties of gel foods, such as a chewy texture, mouthfeel, and associated sensory attributes, for consumers. The results in Table 1 show that a combination of gel strength, breaking force, breaking point, and rigidity of the gel provides as much of a qualitative understanding of the variations of gel properties as any other test [30]. Gellan gum accelerated the gelling speed of gelatin and substantially increased the gel firmness but reduced the color and clarity [27]. The cross-linking formation and turbidity of binary hydrocolloid gels in double helices with monovalent and divalent cation levels should also be considered but not in this study.

The gellan gum was shown to be an effective gelling agent for reducing the syneresis of gelatin gels (Figure 1). Gellan gum was reportedly more effective in a synergistic combination with sweet potato starch in improving syneresis than κ-carrageenan [31]. Agar gel alone has a higher syneresis value than that of gellan gum gel during refrigerated storage. Gelatin had the highest syneresis percentage among these three hydrocolloids and showed a syneresis level of 0.57% after 7 days of refrigeration. Gelatin is a white to cream color powder with a neutral flavor, and it can give the transparency of a solution become cloudy. Gelatin is often used in jellies, mayonnaise, and vinaigrette as a food additive for the food industry. Gelatin–gellan gum gels showed a slight increase to 0.73% in syneresis values after 14 days of refrigeration. The gelatin/gellan gum ratio (0.1/0.4) was shown to have a low syneresis and high breaking force, breaking point, and gel strength during 14 days of storage in a refrigerator in this combination (Figure 1). There seemed to be a strong interaction of the gelatin/gellan gum ratio (0.1/0.4), which resulted in a synergistic effect with a low synersis and high breaking force, breaking point, and gel strength.

Table 1 shows that, in regard to the agar addition, the breaking force and rigidity of the agar–gellan gum mixed gels increased gradually, with an increased agar concentration; however, it increased dramatically to 0.5% using agar alone as opposed to the 0.4/0.1 agar/gellan gum ratio. Gellan gum gel is characterized by its clarity, sparkling, rapid setting behavior, and low concentration for gelling. Gellan gum forms brittle and hard gels. Nevertheless, agar tends to behave as a stiffened and turbid gel when the gel is cooled from the hot dispersion of reversible characteristics. Agar gel demonstrated a higher breaking force, gel strength, and rigidity compared to gellan gum gel and the agar–gellan binary gel system. Agar gel is more brittle and harder than that of gellan gum gel. The solution of agar–gellan binary hydrocolloids on cooling can also form a well-set thermoreversible gel. Such behavior also occurred when 0.1% gelatin was added to 0.4% agar gel. Gellan gum exhibited a more pronounced effect on decreasing the breaking force and rigidity than gelatin at the 0.1% gum concentration, indicating different interactions between agar–gelatin and agar–gellan gum molecules. This can be attributed to the differences in molecular structure and the flexibility of the gum chains. The agar/gellan gum ratio (0.1/0.4) was shown to have low syneresis and a low breaking force, breaking point, gel strength, and rigidity during 14 days of storage in a refrigerator in this combination (Figure 1).

The addition of gellan gum was shown to be a most effective gelling agent in the reduction of syneresis and to increase the breaking point in those binary gels stored in a refrigerator for 7 days (Figure 1). 

### 2.2. Textural Properties and Syneresis Percentage of Combined Use of Modified Corn Starch and Gum Gels

The addition of gellan gum to modified waxy corn starch generally resulted in significant (*p* ≤ 0.05) increases in all the parameters of penetration testing of the modified corn starch/gellan gum mixtures (Table 2). Starch also has been used in processing foods as a thickening material. The gelatinized swollen waxy corn starch consists mainly of amylopectin without the influence of amylose in this study. The retrogradation of waxy corn starch during refrigerated storage was also evaluated. The gelatinization of waxy corn starch in the presence of gellan gum can lead to an increase in viscosity from a rheological point of view. The addition of gellan gum forms gellan-waxy corn starch mixtures with different texture properties, which might also depend on other factors, such as the extent of granular disruption, cationic concentrations, and gellan concentration in the gellan–starch mixture. The use of hydrocolloid–starch mixtures has also improved the water-holding, texture properties, freeze–thaw stability, and prevented the syneresis of gels.

This effect was more pronounced in those mixtures that had an addition of agar to modified corn starch (Table 2). This indicated a phase compatibility between the waxy corn starch and agar components. When a high hardness of the waxy corn starch gel was needed, the method of the addition of agar was more effective and lower in cost. Table 2 shows that, in the penetration testing of modified corn starch/gelatin, all of the parameters increased very slightly, with an increase in gelatin concentrations in the range of 0.1–0.2%. The addition of gelatin and gellan gum to the modified corn starch generally resulted in a dramatic increase in the breaking point (Table 2). This effect was not observed as the concentration of agar increased (Table 2).

**Figure 2:**
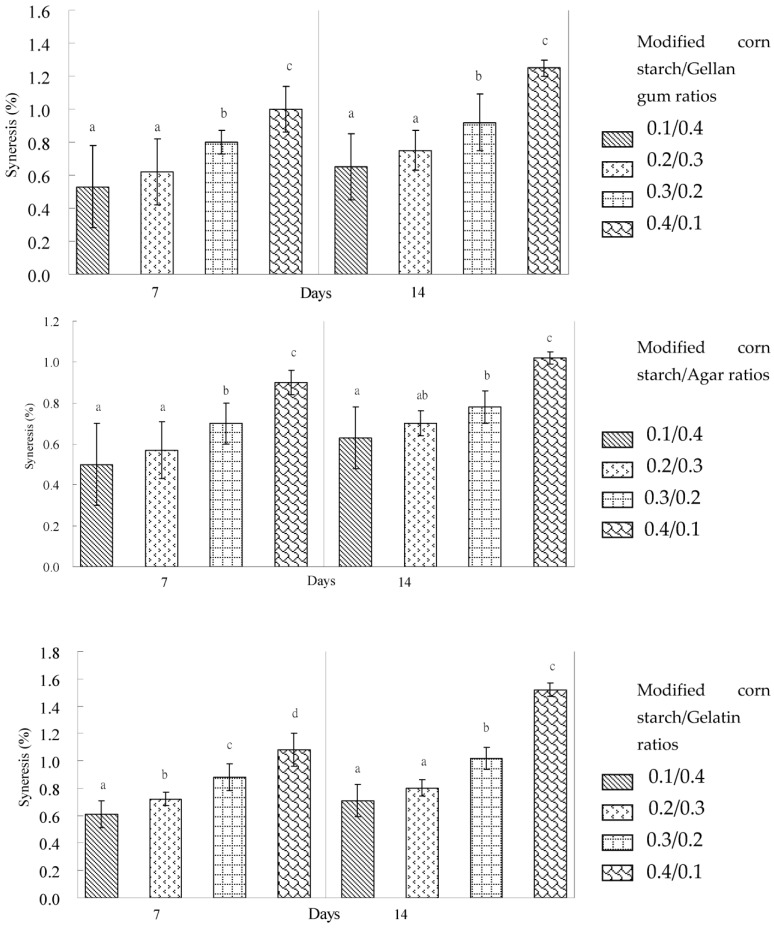
Syneresis of modified corn starch and gum mixed gels stored at 4 °C for 7 days and 14 days. Data with different letters for each storage day represent significant differences at *p* < 0.05.

Indications were that, when the high gel strength and breaking force of the modified corn starch–gum mixtures were required, the addition of agar was efficient in the modified corn starch gels. When a high breaking point of the modified corn starch gel was required, the addition of gellan gum was more appropriate than gelatin or agar. The increase in the syneresis percentage was more obvious for the modified corn starch/gum gel systems than the mixed gum gel systems kept in a refrigerator for 7 and 14 days (Figure 1 and Figure 2). This phenomenon could be caused by an increase in the molecular associations between starch chains—in particular, the retrogradation of amylose [32], which results in the expulsion of water from the mixed gel structure [33]. The results demonstrated that the syneresis percentage of the modified waxy corn starch/gum gel systems was higher than the syneresis percentage of the mixed gum gel systems (Figure 1 and Figure 2). This result showed that the retrogradation of amylopectin from waxy corn starch mainly caused the expulsion of water from the modified waxy corn starch during refrigerated storage. However, the expulsion of water from the mixed gum gel systems also occurred at low levels compared to that of the retrogradation of waxy corn amylopectin.

The effect of hydrocolloids on the syneresis percentage in the modified corn starch gels is presented in Figure 2. When refrigerated for 7 and 14 days, gelatin, gellan gum, and agar at 0.2% and 0.3% levels significantly (*p* ≤ 0.05) reduced the syneresis percentage of the modified corn starch gels. The data showed that modified waxy corn starch with 0.2% to 0.3% gelatin, gellan gum, and agar could improve water retention during refrigerated temperatures for storage. Modified corn starch–gum gel with 0.1% gum had a high syneresis value after 7 and 14 days of refrigeration (Figure 2). Although the cooked-up waxy corn modified starch was developed for improving the freeze–thaw stability and consistent quality at the pH range of 3.5 to 7.5, it has been broadly used as a thickening in fillings, dairy, sauces, and weaning foods. However, modified corn starch with gellan gum and agar displayed a lower syneresis percentage and behaved differently from the gel that contained gelatin. This may be due to the similar chemical structures of waxy corn starch, gellan gum, and agar, leading to the increase in hydrogen bonds and water-holding capacities observed by the syneresis of the binary systems during refrigerated storage. The waxy corn starch underwent the retrogradation of amylopectin during refrigerated storage. Syneresis is an important indicator that is used to evaluate the ability of starch to withstand undesirable physical changes that occur during cold storage and freezing and thawing variations. Hydrocolloids are usually applied to improve rheological properties and the texture of starch-based products [21]. Starch-based products can be modified to have a higher viscosity and thereby reduce syneresis through the use of small quantities of hydrocolloids [34]. Hydrocolloids hinder starch retrogradation and stabilize gelatinized starch during frozen storage [31,35]. Freeze–thawed rice starch–konjac glucomannan mixed gels displayed a markedly lower syneresis percentage and behaved differently from the rice starch gel [36]. The effects of other hydrocolloids on the reduction of syneresis in natural starch have been widely studied [37,38]. It has been claimed that the effects of hydrocolloids in decreasing syneresis are due to the retardation of amylose [35] and an increase in the viscosity of starch paste [31]. The modified corn starch with gellan gum, agar, and gelatin ratios of 0.1/0.4 were shown to have a low syneresis and high breaking force, breaking point, gel strength, and rigidity during 14 days of storage in a refrigerator of the binary gum combined system (Figure 2).

The changes in the syneresis percentage of hydrocolloid gel mixed with gum or with modified corn starch are shown in Figure 1 and Figure 2. In general, the syneresis of the modified corn starch mixed gels decreased with the increased ratio of gum. The main difference was the effect of gellan gum on the mixed gel syneresis reduction and breaking point increase. The syneresis percentage decreased with an increased agar concentration of more than 0.3% (*w*/*w*). The effect of agar on the mixed gel strength and breaking force showed that, with an increase in the concentration of agar, the gel strength and breaking force increased dramatically. In contrast, gelatin did not significantly improve the syneresis of the mixed gels, and its effects were more inconspicuous than gellan gum in this research. There were indications that the addition of gelatin to the mixed gel systems could increase the breaking point of modified corn starch and the agar gels.

## 3. Conclusions

This study demonstrated that agar and gellan gum have good synergistic properties with modified corn starch and that both gums can increase the gel strength, rigidity, and the breaking force of mixed gels. This synergistic interaction was also responsible for the decreasing synersis percentage of the mixed gels. When the mixed gels containing agar were stored in a refrigerator for 7 and 14 days, agar moderately reduced the syneresis percentage and dramatically increased the gel strength and rigidity. Agar increased the breaking force; it had a lower breaking point than other hydrocolloids during penetration testing. The outcome of blending gelatin with other hydrocolloids was positive but less obvious than the textural alternation and syneresis percentage reduction of gellan gum and agar. The reason for the increase in the modified waxy corn starch concentration from 0.1 to 0.4%, with an increased syneresis percentage of the mixed gels after 7 and 14 days of refrigeration, might be due to the reorganization of the amylopectin molecules with more starch and less hydrocolloid added. The syneresis levels of the modified waxy corn starch/gum gel systems were higher than those of the mixed gum gel systems during 14 days of storage in a refrigerator. It showed that the retrogradation of amylopectin from waxy corn starch mainly caused the expulsion of water from the modified waxy corn starch during refrigerated storage. Our results suggest that gellan gum can increase the gel strength, breaking point, and breaking force of mixed gels containing the other hydrocolloids (agar, gelatin, and modified corn starch). The gel strength and breaking force of the modified corn starch–gellan and agar gels decreased with the increased modified corn starch concentration (0 to 0.4%). In all of the experiments, an increase ratio of gum caused a decreased rate of syneresis in the modified corn starch mixed gels. The results could be used as a formula adjustment for ready-to-eat binary gel foods. Food processers could use these results to formulate better textural properties and adjust to consumer likes using the intrinsic interactions between hydrocolloids, modified waxy corn starch, and water, leading to a decrease in syneresis. The results obtained from this work could be used to explain more complex emulsified fat globes with model binary food gels such as milk pudding including milk, sugar, cream, and eggs in the future. A small deformation texture testing, dynamic viscoelasticity, and sensory attribute data collection would be helpful for the fundamental comparisons and explanations of these binary hydrocolloid systems in the future.

## 4. Materials and Methods

### 4.1. Raw Materials

Modified corn starch (CLEARAM^®^ CH 20, Roquette, France) is a pregelatinized drum dried waxy corn starch (E1422) used as an additive for thickening in dairy, fillings, sauces, and weaning foods. Highly acyl gellan gum (KELCOGEL^®^ LT-100, Cpkelco, Chicago, IL, USA), a water-soluble polysaccharide produced by fermentation, pork skin gelatin bloom 200 (VIDOGUM L200, UNIPERKIN, Thiurgau, Switzerland), which has a hot viscosity of 1700–2300 mPa.s., and agar C powder were provided by Gemfont Corporation (Taipei, Taiwan). The agar C powder was extracted from seaweeds gracilaria of different origins.

### 4.2. Preparation of Hydrocolloid Gels

Hydrocolloid mixtures were composed of 1000 g of distilled water and 0.5% *w/w* total hydrocolloid (5 g). The required volume of each polymer solution to give different polymer ratios is given in Table 3. The concentration used in this research was based on the weight of the powder of the mixed gums. Binary hydrocolloid gels were prepared using two kinds of hydrocolloids from gellan gum, gelatin, agar, or modified corn starch at different ratios of hydrocolloid mixtures (0/0.5, 0.1/0.4, 0.2/0.3, 0.3/0.2, 0.4/0.1, and 0.5/0) and water. The mixture was stirred until homogeneous and sterilized using an autoclave (HL-380 Fully Automatic High-Pressure Sterilizer, Hanlein, Taiwan) and heating for 20 min at 100 °C. Aliquots of the samples were poured into glass beakers (20 mL, Φ 21 mm, H 33 mm) and polypropylene cups (100 mL, Φ 70 mm on top, Φ 40 mm at bottom, H 54 mm). The samples were then cooled to room temperature (25–27 °C) and stored in a refrigerator (4–5 °C) for 7 and 14 days prior to their evaluation.

### 4.3. Measurement of Textural Properties

Penetration testing of the hydrocolloid gels was carried out using a Model TA-XT2 texture analyzer (Stable Micro System, Haslemere, UK). Six hydrocolloid gel samples from each mixture were subjected to breaking force (B.F.) and breaking point (B.P.) measurements using a spherical plunger (5 mm diameter) with a depression speed of 100 mm/min. Gel strength (G.S.) and rigidity (R.) were calculated using the following equations [30]:

Gel strength (g × mm) = breaking force (g) × breaking point (mm)
Rigidity (g/mm) = breaking force (g)/breaking point (mm)

Measurements were carried out at room temperature (25–27 °C) in duplicate.

### 4.4. Evaluation of Syneresis

The method suggested by Charoenrein et al. [39] was used with modifications to determine the syneresis of the hydrocolloid gels. All hydrocolloid gels were stored at 4 °C for 7 and 14 days with covers. Before storage, the samples were weighed (w1). The separated water was decanted for 2 min and weighed (w2) again after 1 week or 2 weeks of refrigeration. Syneresis of the hydrocolloid gel was calculated as (w1 − w2)/w1) and expressed as a percentage.

### 4.5. Statistical Analysis

All results were tested using ANOVA. The differences between the means at a 5% significance level (*p* ≤ 0.05) were determined using Duncan’s multiple range test. All of the statistical analyses were performed using the SPSS program, version 12.0 (SPSS Inc., Chicago, IL, USA).

## Figures and Tables

**Table 1:** Effect of binary gum ratios (0.5%) on the textural characteristics ^1^ of mixed gels penetration test (mean ± SD, n = 6).

	Breaking Force	Breaking Point	Gel Strength	Rigidity
	(g)	(mm)	(g × mm)	(g/mm)
Gelatin/Agar ratios				
0/0.5	67.1 ± 2.5 ^f^	2.84 ± 0.08 ^a^	191 ± 6 ^f^	23.71 ± 1.50 ^f^
0.1/0.4	50.1 ± 1.9 ^e^	3.21 ± 0.06 ^b^	161 ± 5 ^e^	15.62 ± 0.81 ^e^
0.2/0.3	40.3 ± 0.8 ^d^	3.87 ± 0.15 ^c^	156 ± 4 ^d^	10.42 ± 0.62 ^d^
0.3/0.2	31.1 ± 1.4 ^c^	4.69 ± 0.28 ^d^	146 ± 5 ^c^	6.75 ± 0.62 ^c^
0.4/0.1	19.7 ± 0.8 ^b^	5.34 ± 0.06 ^e^	105 ± 3 ^b^	3.73 ± 0.26 ^b^
Gelatin/Gellan gum ratios				
0.1/0.4	9.6 ± 0.3 ^e^	9.36 ± 0.48 ^e^	90 ± 3 ^e^	1.04 ±0.08 ^a^
0.2/0.3	9.2 ± 0.2 ^d^	8.75 ± 0.26 ^d^	80 ± 2 ^d^	1.06 ±0.06 ^a^
0.3/0.2	8.8 ± 0.2 ^c^	8.23 ± 0.16 ^c^	72 ± 2 ^c^	1.07 ± 0.05 ^a^
0.4/0.1	8.2 ± 0.1 ^b^	7.25 ± 0.28 ^b^	60 ± 2 ^b^	1.14 ± 0.04 ^b^
0.5/0	7.3 ± 0.3 ^a^	6.10 ± 0.20 ^a^	44 ± 2 ^a^	1.20 ± 0.05 ^b^
Agar/Gellan gum ratios				
0/0.5	10.2 ± 0.4 ^a^	10.2 ± 0.3 ^c^	103 ± 4 ^a^	1.00 ± 0.06 ^a^
0.1/0.4	14.0 ± 0.7 ^b^	8.37 ± 0.35 ^a^	117 ± 2 ^b^	1.68 ± 0.15 ^b^
0.2/0.3	15.3 ± 0.4 ^c^	8.43 ± 0.08 ^a^	129 ± 3 ^c^	1.81 ± 0.06 ^c^
0.3/0.2	16.1 ± 0.6 ^d^	8.52 ± 0.26 ^a^	137 ± 4 ^d^	1.89 ± 0.11 ^cd^
0.4/0.1	18.0 ± 0.3 ^e^	9.32 ± 0.26 ^b^	168 ± 4 ^e^	1.94 ± 0.08 ^d^

^1^ Gel strength = (Breaking force × Breaking point); Rigidity = (Breaking force/Breaking point); Means with different letters in the same sub-columns significantly differ (*p* ≤ 0.05).

**Table 2:** Effect of modified corn starch–gum ratios (0.5%) on the textural characteristics ^1^ of mixed gels penetration test (mean ± SD, n = 6).

	Breaking Force	Breaking Point	Gel Strength	Rigidity
	(g)	(mm)	(g × mm)	(g/mm)
Modified corn starch/Gellan gum ratios				
0/0.5	10.2 ± 0.4 ^e^	10.20 ± 0.3 ^d^	103 ± 4 ^e^	1.00 ± 0.06 ^d^
0.1/0.4	9.3 ± 0.2 ^d^	9.42 ± 0.21 ^c^	87 ± 3 ^d^	0.99 ± 0.04 ^d^
0.2/0.3	8.7 ± 0.2 ^c^	9.37 ± 0.23 ^c^	82 ± 3 ^c^	0.94 ± 0.02 ^c^
0.3/0.2	7.7 ± 0.2 ^b^	8.83 ± 0.20 ^b^	68 ± 2 ^b^	0.88 ± 0.04 ^b^
0.4/0.1	6.1 ± 0.2 ^a^	7.45 ± 0.21 ^a^	46 ± 2 ^a^	0.83 ± 0.03 ^a^
Modified corn starch/Agar ratios				
0/0.5	67.1 ± 2.5 ^f^	2.84 ± 0.08 ^e^	191 ± 6 ^e^	23.71 ± 1.50 ^d^
0.1/0.4	58.1 ± 1.7 ^d^	2.54 ± 0.06 ^d^	147 ± 5 ^d^	22.91 ± 0.92 ^c^
0.2/0.3	49.3 ± 1.2 ^c^	2.12 ± 0.07 ^c^	104 ± 5 ^c^	23.20 ± 0.82 ^c^
0.3/0.2	40.1 ± 0.8 ^b^	1.88 ± 0.06 ^b^	75 ± 3 ^b^	21.45 ± 0.81 ^b^
0.4/0.1	29.4 ± 1.0 ^a^	1.49 ± 0.02 ^a^	43 ± 2 ^a^	19.70 ± 0.50 ^a^
Modified corn starch/Gelatin ratios				
0/0.5	7.3 ± 0.3 ^b^	6.10 ± 0.20 ^b^	44 ± 2 ^b^	1.20 ± 0.05 ^c^
0.1/0.4	7.3 ± 0.2 ^b^	6.35 ± 0.21 ^b^	46 ± 2 ^b^	1.16 ± 0.01 ^b^
0.2/0.3	7.2 ± 0.2 ^b^	6.23 ± 0.23 ^b^	45 ± 1 ^b^	1.16 ± 0.08 ^b^
0.3/0.2	6.4 ± 0.1 ^a^	5.97 ± 0.10 ^a^	38 ± 1 ^a^	1.08 ± 0.03 ^a^
0.4/0.1	6.2 ± 0.2 ^a^	5.93 ± 0.12 ^a^	37 ± 2 ^a^	1.06 ± 0.03 ^a^

^1^ Gel strength = (Breaking force × Breaking point); Rigidity = (Breaking force/Breaking point); Means with different letters in the same sub-columns significantly differ (*p* ≤ 0.05).

**Table 3:** Experimental designs of the different mixtures of composite gels.

Composite Gels	Modified Corn Starch	Gelatin	Agar	Gellan
	(% *w*/*w*)	(% *w*/*w*)	(% *w*/*w*)	(% *w*/*w*)
Gelatin/agar		0	0.5	
		0.1	0.4	
		0.2	0.3	
		0.3	0.2	
		0.4	0.1	
Gelatin/gellan		0.1		0.4
		0.2		0.3
		0.3		0.2
		0.4		0.1
		0.5		0
Agar/gellan			0	0.5
			0.1	0.4
			0.2	0.3
			0.3	0.2
			0.4	0.1
Modified corn starch/gellan	0.1			0.4
	0.2			0.3
	0.3			0.2
	0.4			0.1
Modified corn starch/agar	0.1		0.4	
	0.2		0.3	
	0.3		0.2	
	0.4		0.1	
Modified corn starch/gelatin	0.1	0.4		
	0.2	0.3		
	0.3	0.2		
	0.4	0.1		

## Data Availability

The data presented in this work are available on request from the corresponding author.

## References

[1] Mahmood K., Kamilah H., Shang P.L., Sulaiman S., Ariffin F., Alias A.K. (2017). A review: Interaction of starchy non-starch hydrocolloid blending and the recent food applications. Food Biosci..

[2] Fonkwe L., Narsimhan G., Cha A.S. (2003). Characterization of gelation time and texture of gelatin and gelatin-polysaccharide mixed gels. Food Hydrocoll..

[3] Walayat N., Liu J., Nawaz A., Aadil R.M., Lopez-Pedrouso M., Lorenzo J.M. (2022). Role of Food hydrocolloids as antioxidants along with modern processing techniques on the surimi protein gel textural properties. Antioxidants.

[4] Zasypkin D.V., Dumay E., Cheftel J.C. (1996). Pressure- and heat- induced gelation of mixed β-lactoglobulin/xanthan solutions. Food Hydrocoll..

[5] Sworn G., Stouby L., Phillips G.O., Williams P.A. (2021). Gellan gum. Hook of Hydrocolloids.

[6] Chandrasekaran R., Radha A. (1995). Molecular architectures and functional properties of gellan gum and related polysaccharides. Trends Food Sci. Technol..

[7] Li A., Gong T., Li X., Guo Y. (2020). Preparation of thermally stable emulsion gels based on glucono-δ-lactone induced gelation of gellan gum. Int. J. Bio. Macromol..

[8] Sanderson G.R., Bell V.L., Clark R.C., Ortega D., Phillips G.O., William P.A., Wedlock D.J. (1988). The texture of gellan gum. Gums and Stabilizers for the Food Industry 4.

[9] Ge H., Wu Y., Woshnak L.L., Mitmesser S.H. (2021). Effects of hydrocolloids, acids and nutrients on gelatin network in gummies. Food Hydrocoll..

[10] Morris V.J. (1990). Starch gelation and retrogradation. Trends Food Sci. Technol..

[11] Oakenfull D., Pearce J., Burley R.W., Damodaran S., Paraf A. (1997). Protein gelation. Food Protein and Their Applications.

[12] Qin Y. (2018). Seaweed hydrocolloids as thickening, gelling, and emulsifying agents in functional food products. Bioactive Seaweeds for Food Applications.

[13] Stanley N.F., Stephen A.M. (1995). Agars. Food Polysaccharides and Their Application.

[14] Harris P. (1990). Food Gels.

[15] Labropoulos K.C., Niesz D.E., Danforth S.C., Kevrekidis P.G. (2002). Dynamic rheology of agar gels: Theory and experiments. Part I. Development of a rheological model. Carbohydr Polym..

[16] Ross K.A., Pyrak-Nolte L.J., Campanella O.H. (2006). The effect of mixing conditions on the material properties of an agar gel-microstructural and macrostructural considerations. Food Hydrocoll..

[17] Ikeda S., Nitta Y., Kim B.S., Temsiripong T., Pongsawatmanit P., Nishinari K. (2004). Single-phase mixed gels of xyloglucan and gellan. Food Hydrocoll..

[18] Tolstoguzov V.B. (1995). Some physico-chemical aspects of protein processing in foods. Multicomponent gels. Food Hydrocoll..

[19] BeMiller J.N. (2011). Pasting, paste, and gel properties of starch-hydrocolloid combinations. Carbohydr. Polym..

[20] Kim H.S., BeMiller J.N. (2012). Effects of hydrocolloids on the pasting and paste properties of commercial pea starch. Carbohydr. Polym..

[21] Shi X., BeMiller J.N. (2002). Effects of food gum and viscosities of starch suspension during pasting. Carbohydr. Polym..

[22] Sodini I., Remeuf F., Haddad S., Corrieu G. (2004). The relative effect of milk base, starter, and process on yogurt texture: A review. Crit. Rev. Food Sci. Nutr..

[23] Sudhakar V., Singhal R.S., Kulkarni P.R. (1995). Effect of sucrose on starch-hydrocolloid interactions. Food Chem..

[24] Lal S.N.D., O’Connor C.J., Eyres L. (2006). Application of emulsifiers in dairy products of high rheology. Adv. Colloid Interface Sci..

[25] Supavititpatana P., Wirjantoro T.I., Apichartsrangkoon A., Raviyan P. (2008). Addition of gelatin enhanced gelation of corn-milk yogurt. Food Chem..

[26] Karim A.A., Norziah M.H., Seow C.C. (2000). Methods for the study of starch retrogradation. Food Chem..

[27] Schrieber R., Gareis H. (2007). Gelatine Handbook.

[28] Papageorgiou M., Kasapis S., Richardson R.K. (1994). Steric exclusion phenomena in gellan/gelatin systems I. Physical properties of single and binary gels. Food Hydrocoll..

[29] Mao R., Tang J., Swanson B.G. (2000). Texture properties of high and low acyl mixed gellan gels. Carbohydr. Polym..

[30] Tang Q., McCarthy O.J., Munro P.A. (1995). Effect of pH on whey protein concentrate gel properties: Comparisons between small deformation (Dynamic) and large deformation (Failure) testing. J. Texture Stud..

[31] Lee M.H., Baek M.H., Cha D.S., Park H.J., Lim S.T. (2002). Freeze-thaw stabilization of sweet potato starch gel by polysaccharide gums. Food Hydrocoll..

[32] Morrison N.A., Clark R.C., Chen Y.L., Talashek T., Sworn G., Nishinari K., Kremer F., Lagaly G. (1999). Gelatin alternatives for the food industry. Physical Chemistry and Industrial Application of Gellan Gum.

[33] Saartratra S., Puttanlekb C., Rungsardthong V., Uttapap D. (2005). Paste and gel properties of low-substituted acetylated canna starches. Carbohydr. Polym..

[34] Mali H., Ferrero C., Redigonda V., Beleia A.P., Grossmann M.V.E., Zaritzky N.E. (2003). Influence of pH and hydrocolloids addition on yam (*Dioscorea alata*) starch pastes stability. LWT Food Sci. Technol..

[35] Ferrero C., Martino M.N., Zaritzky N.E. (1994). Corn starch-xanthan gum interaction and its effect on the stability during storage of frozen gelatinized suspensions. Starch/Starke.

[36] Charoenrein S., Tatirat O., Rengsutthi K., Thongngam M. (2011). Effect of konjac glucomannan on syneresis, textural properties and the microstructure of frozen rice starch gels. Carbohydr. Polym..

[37] Muadklay J., Charenrein S. (2008). Effects of hydrocolloids and freezing rates on freeze-thaw stability of tapioca starch gels. Food Hydrocoll..

[38] Weber F.H., Queiroz F.P.C., Chang Y.K. (2008). Freeze-thaw stability of normal, waxy and high amylase corn starch gels with added guar and xanthan gums. Food Sci. Technol..

[39] Charoenrein S., Tatirat O., Muadklay J. (2008). Use of centrifugation-filtration fordetermination of syneresis in freeze-thaw starch gels. Carbohydr. Polym..

